# Invasive *Salmonella* Discussed in Africa Consensus Meeting 2014, Blantyre, Malawi

**DOI:** 10.3201/eid2111.150624

**Published:** 2015-11

**Authors:** Robert S. Heyderman, John A. Crump, on behalf of the conference attendees

**Affiliations:** Author affiliations: Malawi-Liverpool-Wellcome Trust Clinical Research Programme, University of Malawi, Blantyre, Malawi (R.S. Heyderman); Centre for International Health, University of Otago, Dunedin, New Zealand (J.A. Crump)

**Keywords:** *Salmonella* *enterica*, typhoid, Africa, nontyphoidal salmonella, vaccine, bacteria

*Salmonella enterica* is a leading cause of invasive bacterial disease among adults and children in Africa (*1*). Other prominent invasive pathogens such as *Haemophilus influenzae* type b (Hib), *Neisseria meningitidis* serogroup A, and *Streptococcus pneumoniae* are combated by highly effective protein-conjugate vaccines; therefore, life-threatening disease caused by invasive *Salmonella* infection is likely to become a more prominent disease control priority.

Although typhoid fever was described in detail by William Budd in the 1800s, invasive *Salmonella* infection (either typhoid fever or invasive nontyphoidal *Salmonella*) has not been seen as a public health priority in Africa. At a workshop on typhoid fever held at the Pan American Health Organization in 1984 (*2*), it was reported that there was “a sense of optimism among participants for improved, worldwide control of typhoid fever.” Although 30 years later, there has been progress in the development of typhoid bacterial vaccines, the control of invasive *Salmonella* in Africa still seems remote. Many in the clinical and public health communities cling to the established ideology that clinical illness caused by nontyphoidal *Salmonella* in Africa is mostly self-limited enterocolitis and that animals are a likely reservoir; and it is frequently argued that since typhoid fever is associated with a relatively low mortality rate, there is a low burden of illness (BOI). Regarding African populations in which invasive nontyphoidal *Salmonella* (iNTS) and typhoidal *Salmonella* frequently co-exist, as summarized in 2 recent articles (*3*,*4*), we are still largely ignorant of the BOI, potential risk factors, routes of transmission, epidemiologic trends, and reservoirs of infection.

To address these knowledge gaps related to invasive *Salmonella* in Africa, at the end of 2014, the Wellcome Trust and the Bill & Melinda Gates Foundation sponsored a consensus meeting of experts in Blantyre, Malawi. We hope that in the future, this meeting will be seen as a landmark event that initiated new collaborations between multidisciplinary teams across the continent. At the meeting, new data were presented from 17 political entities in Africa (Figure). Overview presentations from Melita Gordon (University of Liverpool, UK and the Malawi-Liverpool Wellcome Trust, Malawi) and Florian Marks (International Vaccine Institute, Typhoid Surveillance in sub-Saharan Africa Program, South Korea) emphasized the dynamic nature of invasive *Salmonella* epidemiology in Africa and the extraordinarily high childhood case-fatality ratios for iNTS disease (a term adopted by consensus at the meeting). New iNTS disease BOI estimates were presented that had been calculated through an initiative led by John Crump (University of Otago, New Zealand), suggesting very high incidence in countries of sub-Saharan Africa that mostly affected infants, young children, and young adults. The case-fatality ratio established by expert opinion and review of large case series was estimated at 20%. Together, these findings establish iNTS disease as a major and underappreciated cause of child and adult deaths in Africa. 

**Figure:**
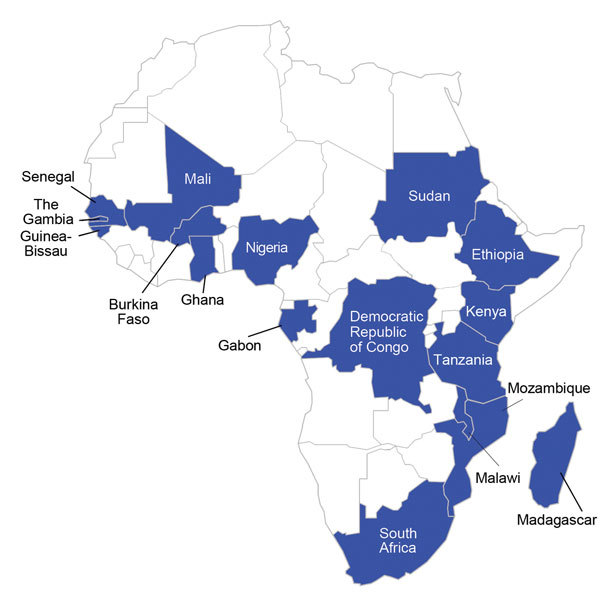
Countries from which bacteremia data were presented at the Invasive *Salmonella* in Africa Consensus Meeting 2014 in Blantyre, Malawi (blue shading).

Although these estimates highlight the paucity of good quality surveillance data from the continent for *Salmonella* in general, data brought to the meeting by Martin Antonio (Medical Research Council Unit, The Gambia), Myron Levine (University of Maryland, United States, and Center for Vaccine Development, Mali), Stephen Obaro (University of Nebraska Medical Center, United States and institutions in Nigeria), Anthony Scott (Kenya Medical Research Institute–Wellcome Trust Research Programme, Kenya), Jan Jacobs (Institute of Tropical Medicine, Belgium and Democratic Republic of Congo), Nick Feasey (Liverpool School of Tropical Medicine, United Kingdom, and the Malawi-Liverpool-Wellcome Trust Programme, Malawi), Quique Bassat (Barcelona Centre for International Health Research and the Manhiça Health Research Centre, Mozambique), Karen Keddy (National Institute for Communicable Diseases, South Africa), and Calman MacLennan (Novartis Institute for Global Health, Italy) showed the value of using multicenter vaccine trials, demographic surveillance sites, and facility-based sentinel surveillance to enhance our BOI information. Myron Levine highlighted how iNTS serovars appear to fluctuate in prominence over time but that most iNTS disease in Africa is caused by *Salmonella* Typhimurium, *Salmonella* Enteritidis, and *Salmonella* Dublin. He also pointed to the typically “spotty” or focal distribution of invasive *Salmonella* that render disease estimates problematic, even within countries. Antimicrobial drug resistance was identified as a likely threat to effective case management. 

Barbara Mahon (Centers for Disease Control and Prevention, United States) reported widespread multidrugresistant, extended-spectrum β-lactamase–producing *Salmonella* Typhimurium in western Kenya, which has only been identified sporadically in other countries in the region (*5*). Data presented from across the continent demonstrated the complicated “magic roundabout” of risk factors for iNTS disease, including malaria, malnutrition, and HIV. Melita Gordon showed how structural equation modeling can be used to unravel the complex relationships between these factors. Ginny Pitzer (Yale School of Public Health, United States) used typhoid data from low and high transmission settings to show how predictive mathematical modeling can help explore the impact of chronic carriage, subclinical infections, and natural immunity on vaccine effectiveness. 

Although many in the audience suspected that human-to-human transmission is key to the biology of iNTS in Africa, novel approaches were discussed that may identify additional environmental and animal reservoirs. Sam Kariuki (Kenya Medical Research Institute, Kenya), Kudakwashe Magwedere (Department of Agriculture, South Africa) and Nigel French (Massey University, New Zealand) discussed the complexities of finding an animal source for iNTS–associated serovars, which are crucial because these represent additional potential targets for intervention. Sharon Tennant (University of Maryland, United States) reviewed the growing armamentarium of molecular and immunological tools now available to revisit the population epidemiology of iNTS disease and typhoid in Africa. This is critical given the sample sizes required for vaccine efficacy trials (≈9,000 persons) and the long timelines (2025–2035) for the development of some *Salmonella* vaccines set out by Allan Saul (Novartis Institute for Global Health, Italy).

To conclude, the group discussed the development of common strategies for diagnosis, antimicrobial treatment, case management, and vaccine and nonvaccine interventions that will address invasive *Salmonella* in Africa. However, it was strongly argued that further population-based studies are needed to better define natural immunity and seroepidemiology, the reservoirs and sources of these pathogens, acquisition and chronic carriage, and in relation to iNTS, the role of the food chain, water, and animals. Through advances in high-throughput whole genome sequencing described by Alison Mather (Wellcome Trust Sanger Institute, United Kingdom) and the analysis of very large datasets of bacterial genes and human clinical phenotypic information, it is hoped that these complex host-pathogen relationships can be further resolved to assist in the modeling of the gaps in our understanding. Finally, Imran Khan (Sabin Vaccine Institute, Coalition Against Typhoid, United States) highlighted the need for advocacy at the policymaking and funding levels to improve the visibility of these neglected diseases.

**Table:** Delegate list: Invasive *Salmonella* in Africa Consensus Meeting 2014, Blantyre, Malawi November 18–19, 2014

Delegate	Institution	Country
William Alexander	Enteric & Hepatic Diseases Branch, National Institute of Allergy and Infectious Disease	United States
Martin Antonio	Medical Research Council Unit, The Gambia	Gambia
Quique Bassat	Barcelona Centre for International Health Research	Spain
	Manhiça Health Research Center	Mozambique
Christoph Blohmke	Oxford Vaccine Group, University of Oxford	United Kingdom
Robert Breiman	Emory Global Health Institute, Emory University	United States
Debbie Burgess	Bill & Melinda Gates Foundation	United States
Fred Cassels	Enteric & Hepatic Diseases Branch, National Institute of Allergy and Infectious Disease	United States
Geoffrey Chipungu	US Centers for Disease Control and Prevention, Malawi Country Office	Malawi
John Crump	University of Otago	New Zealand
Brigitte Denis	Malawi-Liverpool-Wellcome Trust, Clinical Research Programme	Malawi
Zoey Diaz	Bill & Melinda Gates Foundation	United States
Dean Everett	University of Liverpool/ Malawi-Liverpool-Wellcome Trust, Clinical Research Programme	Malawi
Nick Feasey	Liverpool School of Tropical Medicine	United Kingdom
	Malawi-Liverpool-Wellcome Trust, Clinical Research Programme	Malawi
Nigel French	Massey University	New Zealand
Melita Gordon	University of Liverpool/ Malawi-Liverpool-Wellcome Trust, Clinical Research Programme	United Kingdom
Robert Heyderman	Malawi-Liverpool-Wellcome Trust, Clinical Research Programme	Malawi
Jan Jacobs	Institute of Tropical Medicine, Antwerp	Belgium
	Institute of Tropical Medicine	Democratic Republic of Congo
Storn Kabuluzi	Malawi Ministry of Health	Malawi
Ndaru Kaluwa	Malawi-Liverpool-Wellcome Trust, Clinical Research Programme	Malawi
Sam Kariuki	Kenya Medical Research Institute	Kenya
Karen Keddy	National Institute for Communicable Diseases	South Africa
Neil Kennedy	University of Malawi College of Medicine	Malawi
Imran Khan	Sabin Vaccine Institute, Coalition Against Typhoid	United States
Myron Levine	University of Maryland School of Medicine	USA
	Center for Vaccine Development	Mali
Cal MacLennan	Novartis Vaccines Institute for Global Health	Italy
Kudakwashe Magwedere	Department of Agriculture, South Africa	South Africa
Barbara Mahon	US Centers for Disease Control and Prevention	United States
Jane Mallewa	University of Malawi College of Medicine	Malawi
Florian Marks	International Vaccine Institute, Typhoid Surveillance in sub-Saharan Africa Program	South Korea
Pietro Mastroeni	University of Cambridge	United Kingdom
Alison Mather	Wellcome Trust Sanger Institute, Cambridge	United Kingdom
Alastair McGregor	Hospital for Tropical Diseases	United Kingdom
Gretchen Meller	Bill & Melinda Gates Foundation	United States
Mwapatsa Mipando	University of Malawi College of Medicine	Malawi
Chisomo Msefula	University of Malawi College of Medicine	Malawi
Florida Muro	Kilimanjaro Christian Medical University College	Tanzania
Tonney Nyirenda	Malawi-Liverpool-Wellcome Trust, Clinical Research Programme	Malawi
Mulinda Nyirenda	Ministry of Health and Queen Elizabeth Central Hospital	Malawi
Stephen Obaro	University of Nebraska Medical Center United States	United States
Fran Olgemoeller	Malawi-Liverpool-Wellcome Trust, Clinical Research Programme	Malawi	
Ginny Pitzer	Yale School of Public Health	United States	
Hugh Reyburn	London School Hygiene Tropical Medicine	United Kingdom	
Nimako Sarpong	Kumasi Center for Collaborative Research (KCCR)	Ghana	
Allan Saul	Novartis Vaccines Institute for Global Health (NVGH)	Italy	
Anthony Scott	KEMRI-Wellcome Trust Research Programme	Kenya	
Duncan Steele	Bill & Melinda Gates Foundation	United States	
Sharon Tennant	Center for Vaccine Development, University of Maryland Baltimore	United States	
Mike Turner	Wellcome Trust	United Kingdom	
Alison Wallace	Wellcome Trust	United Kingdom	
Anita Zaidi	Bill & Melinda Gates Foundation	United States	
